# LncRNA GUSBP5-AS promotes EPC migration and angiogenesis and deep vein thrombosis resolution by regulating FGF2 and MMP2/9 through the miR-223-3p/FOXO1/Akt pathway

**DOI:** 10.18632/aging.102904

**Published:** 2020-03-10

**Authors:** Li-Li Sun, Feng-Rui Lei, Xu-Dong Jiang, Xiao-Long Du, Lun Xiao, Wen-Dong Li, Xiao-Qiang Li

**Affiliations:** 1 Department of Vascular Surgery, The Second Affiliated Hospital of Soochow University, Suzhou, Jiangsu, China; 2 Department of Vascular Surgery, The Affiliated Drum Tower Hospital, Nanjing University Medical School, Nanjing, Jiangsu, China

**Keywords:** non-coding RNA, endothelial progenitor cells, deep vein thrombosis, angiogenesis, migration

## Abstract

Long non-coding RNAs (lncRNAs) play an essential role in multitudinous physiological and pathological processes, including vascular disease. We previously showed that lncRNA *GUSBP5-AS* (enst00000511042) is upregulated in endothelial progenitor cells (EPCs) of deep veni thrombosis (DVT) patients. Here, we investigate the role and mechanism of *GUSBP5-AS* in EPCs and DVT. Using the DVT model, we found that *GUSBP5-AS* significantly reduced the thrombus size and weight and enhanced the homing ability of EPC to DVT sites to promote resolution and recanalization of thrombus. *GUSBP5-AS* promoted cell cycle progression, proliferation, migration and invasion in EPCs, enhanced EPC angiogenesis *in vitro* and *in vivo*, and inhibited apoptosis. Strikingly, this study showed that *GUSBP5-AS* was unbalanced and modulated Forkhead Box Protein O1 (FOXO1) in EPCs in patients with DVT by interacting with *miR-223-3p*. Mechanistically, *GUSBP5-AS* functions as a sponge of *miR-223-3p*, which targets FOXO1. Both *GUSBP5-AS* knockdown and *miR-223-3p* overexpression remarkably inhibited angiogenesis, migration and invasion in EPCs. Additionally, our data suggested that *GUSBP-AS* activated the Akt pathway and enhanced fibroblast growth factor 2 (FGF2), matrix metalloproteinase-2/9 (MMP2/9) and F-actin expression. Taken together, this study indicates that *GUSBP5-AS* modulates angiogenesis, proliferation and homing ability of EPCs via regulating FGF2 and MMP2/9 expression through the *miR-223-3p*/FOXO1/Akt pathway, which may provide a new direction for the development of DVT therapeutics.

## INTRODUCTION

Deep vein thrombosis (DVT) is a blood clot that forms within a deep vein, especially in leg veins such as the femoral vein. The prevalence of DVT is slightly higher in men than in women [1] and increases with age [2]. Approximately 20%–50% of patients after a first DVT event suffer from post-thrombotic syndrome (PTS), which involves intractable edema, pain, a sensation of heaviness, pigmentation, and even venous ulcers in severe cases [1, 3]. In addition, DVT can be potentially life-threatening when a blood clot breaks off and develops into pulmonary embolism (PE), leading to sudden cardiovascular collapse and death [4, 5]. DVT and PE form a single disease process named venous thromboembolism (VTE), which is the third most common vascular disease in the United States [6, 7]. The main consequences of DVT are death, recurrence, PTS, and major bleeding caused by anticoagulation [5, 8]. Consequently, the quality of life of patients with DVT is severely affected, especially when PTS is present [5, 9]. Approximately 900,000 new cases of DVT are estimated each year in the United States alone, with a mortality rate close to 300,000 cases per year [10, 11]. Therefore, it is important and necessary to study the molecular mechanism of DVT and to explore alternative therapies.

Neovascularization is a major event for the resolution of thrombus [12, 13]. A growing body of data has suggested that bone marrow-derived circulating endothelial progenitor cells (EPCs) are recruited into the thrombus and accelerate thrombus resolution and also have an important role in physiological and pathological neovascularization in adults [13–16]. In addition, EPCs have the ability to proliferate, migrate, and form new blood vessels through differentiation into endothelial cells, and have recently become a promising therapeutic approach for DVT-related thrombus resolution in patients who have achieved limited success with current treatment options [13, 16, 17]. Although EPCs show good therapeutic effects, their clinical application still faces many challenges. The number and function of circulating EPCs can be affected by adverse conditions in the microenvironment, including advanced age, diabetes, cardiovascular risk factors, ischemic disease, and graft vasculopathy [18–21]. Therefore, the development of methods to improve the recruitment of EPCs to the thrombus and enhance angiogenesis is needed.

Long non-coding RNAs (lncRNAs), a class of non-coding RNAs longer than 200 nucleotides, regulate molecules through multiple mechanisms including epigenetic modification, as well as transcription and translation regulation [22, 23]. Studies have also shown that lncRNAs can serve as competitors or reservoirs of miRNAs to indirectly regulate gene expression [24], and lncRNAs are emerging as crucial regulators of angiogenesis, development, differentiation, metabolism, and autophagy [21, 25–28]. Autophagy, one of the crucial degradation pathways in eukaryotes, is important for maintaining cellular homeostasis and is associated with vascular diseases [29]. Furthermore, multiple lncRNAs are dysregulated in various diseases including vascular disease and may control disease progression, providing promising new targets for the treatment of vascular diseases [30, 31]. However, the roles of lncRNAs in DVT recanalization and resolution are still largely unknown.

We previously performed a comprehensive expression profiling analysis of lncRNAs in DVT patients, and found that lncRNA *GUSBP5-AS* (enst00000511042) was significantly upregulated in EPCs from DVT patients compared with healthy controls [32]. *GUSBP5-AS* is located at chromosome 4q31.21 on the somatic map with a length of 342 bp. We suspected that *GUSBP5-AS* may modulate angiogenesis in EPCs and participate in the dissolution of thrombi. This article focuses on the role of *GUSBP5-AS* in EPCs and in DVT resolution, and explores the underlying molecular mechanism. These findings may contribute to the development of a novel effective treatment for DVT.

In this study, we demonstrated that *GUSBP5-AS* was upregulated in EPCs in DVT patients. Overexpression of *GUSBP5-AS* in EPCs significantly reduced thrombus size and weight and facilitated the homing ability of EPCs to DVT sites to promote DVT recanalization and resolution. Furthermore, we found that *GUSBP5-AS* promotes angiogenesis and cell proliferation, migration, and invasion, and inhibits apoptosis in EPCs by modulating fibroblast growth factor 2 (FGF2) and matrix metalloproteinase-2/9 (MMP2/9) expression through the *miR-223-3p*/FOXO1 (Forkhead Box Protein O1)/Akt pathway. These findings may provide new insights into the molecular mechanisms of lncRNAs in regulating angiogenesis and suggest a potential novel therapeutic approach for DVT.

## RESULTS

### *GUSBP5-AS* facilitates EPC homing to thrombosis sites and DVT recanalization and resolution

**Figure 1:**
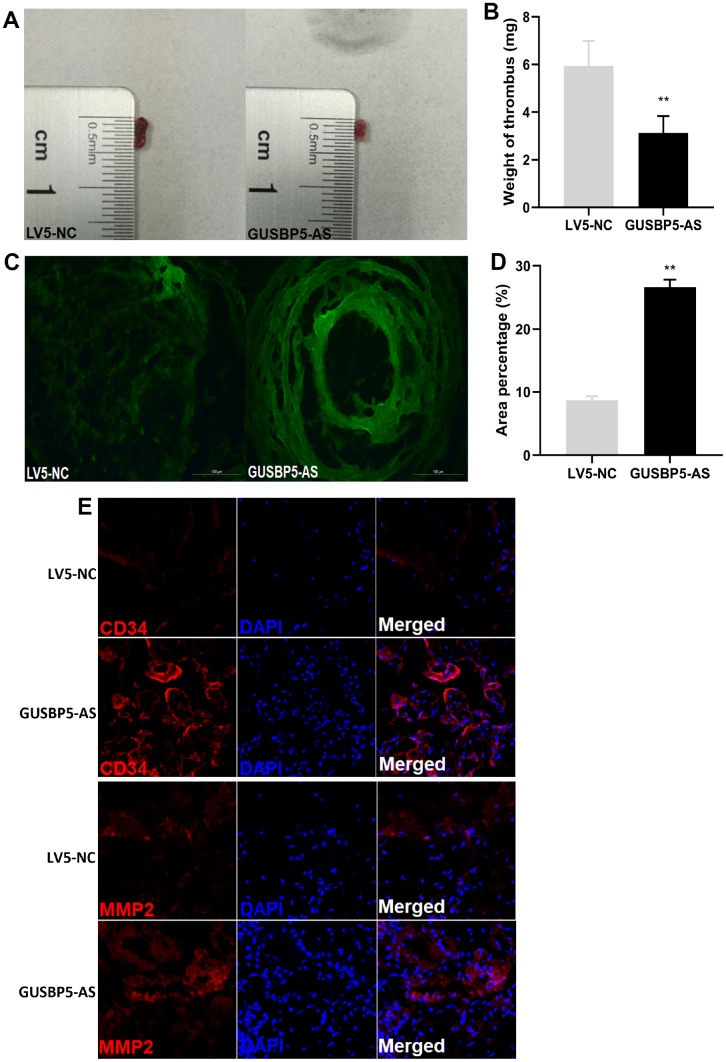
**Effects of lncRNA *GUSBP5-AS* on the treatment of DVT.** (**A**) Representative images of the thrombosis in GFP-*GUSBP5-AS*-EPC group and LV5-NC group were observed. (**B**) Weight of the venous thrombi at day 7 after the transplantation. Data are expressed as mean ± SEM (n = 8 mice per group), **P < .01 compared to the corresponding negative controls. (**C**) Representative images of recruitment of GFP-positive EPCs in DVT (×200). (**D**) Percentage of green fluorescent area in DVT. **P < .01. (**E**) The expression levels of CD34 and MMP2 in thrombosis were detected by immunofluorescence.

EPCs can be recruited to sites of thrombosis and accelerate vasculogenesis and thrombosis resolution [33]. We next investigated whether *GUSBP5-AS* regulates the homing ability of EPCs to thrombotic sites and participates in DVT recanalization and resolution. We constructed a model of jugular vein thrombosis in nude mice and injected GFP-*GUSBP5-AS*-EPCs or GFP-NC-EPCs, as a control, to thrombi. The thrombosis size in the GFP-*GUSBP5-AS*-EPC group was smaller than that in the LV5-NC group (Figure 1A). In the *GUSBP5-AS* overexpression group, the thrombus weight was significantly reduced on day 7 after transplantation compared with the LV5-NC group (Figure 1B). In addition, the number of EPCs homing to thrombosis sites in the *GUSBP5-AS*-overexpressing EPC group was higher than that of the LV5-NC group (Figure 1C–1D). Immunofluorescence analysis demonstrated that there were higher expression levels of CD34, a marker of angiogenesis in the thrombus, and MMP2, an essential regulator of early thrombolysis, and more nucleated cells including EPCs in the thrombi of the *GUSBP5-AS* overexpression group compared with the LV5-NC group (Figure 1E). Together, these results indicated that *GUSBP5-AS* accelerates DVT revascularization and resolution and may represent a novel potential target for DVT treatment.

### *GUSBP5-AS* enhances the angiogenesis of EPCs *in vivo* and *in vitro,* migration, invasion, F-actin filaments and proliferation, and inhibits cell apoptosis

**Figure 2:**
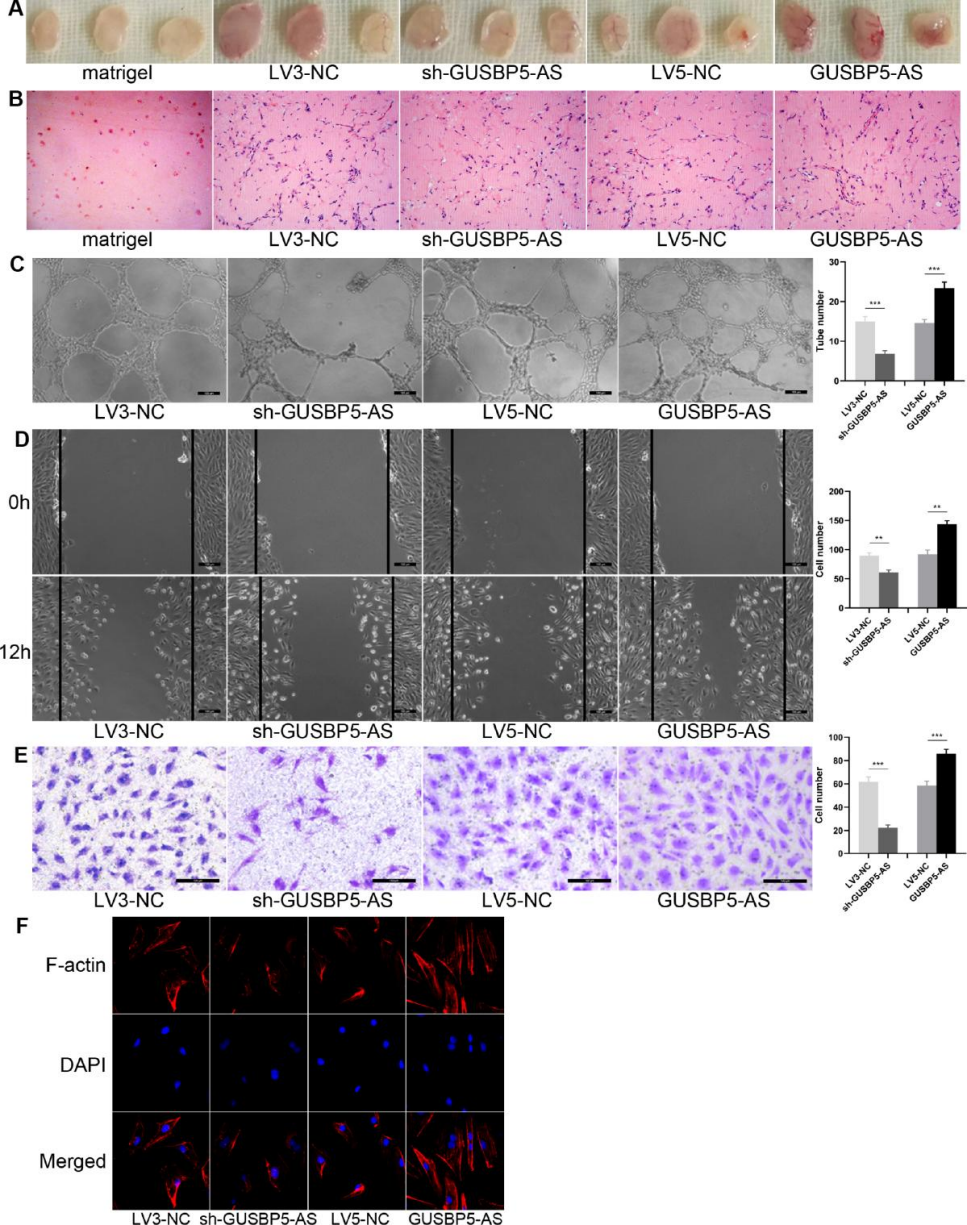
**Effects of lncRNA *GUSBP5-AS* on angiogenesis of EPCs *in vivo and in vitro*, EPC migration and invasion.** (**A**) In vivo angiogenesis was evaluated at Day 7 after subcutaneous injection of Matrigel-mixed EPCs into nude mice. (**B**) HE staining: lncRNA *GUSBP5-AS* knockdown and overexpression, respectively, decreased and increased tube formation by EPCs in vivo (original magnification, ×200). (**C**) In vitro tube formation assay. Scale bar=100μm. (original magnification, ×100). LncRNA *GUSBP5-AS* knockdown and overexpression, respectively, decreased and increased tube number. ***P< .001. (**D**) Wound healing assay showing the effects of lncRNA *GUSBP5-AS* on EPC migration. Scale bar=100μm. (original magnification, ×100). LncRNA *GUSBP5-AS* knockdown and overexpression, respectively, decreased and increased cell migration in EPCs. **P< .01. (**E**) Effects of lncRNA *GUSBP5-AS* on cell invasion ability were analysed. Scale bar=100μm. (original magnification, ×200). Data are represented as mean ± SEM. ***P< .001. (**F**) Effect of lncRNA *GUSBP5-AS* on actin cytoskeleton structure in cultured EPCs. Cells were fixed, permeabilized, stained with rhodamine–phalloidin and DAPI, and visualized by confocal microscopy; down-regulation of *GUSBP5-AS* impaired F-actin while *GUSBP5-AS* overexpression prevented disruption of actin cytoskeletal structure.

To examine the effects of *GUSBP5-AS* on EPCs, we performed gene knockdown and overexpression by lentiviral infection and observed EPC angiogenesis *in vivo* and *in vitro*, migration and invasion. The results of angiogenesis *in vivo* assay showed that implants with *GUSBP5-AS*-overexpressing EPCs were redder in appearance than the LV5-NC group, while the implants with *GUSBP5-AS* knockdown EPCs were less red compared with those of the LV3-NC groups (Figure 2A). No differences were observed between the LV5-NC group and LV3-NC group, and the Matrigel alone group (without cells) was not red in appearance. H&E staining revealed more luminal structures in implants with *GUSBP5-AS*-overexpressing EPCs than in those with LV5-NC EPC group and fewer luminal structures in implants with *GUSBP5-AS*-knockdown EPCs than in in those LV3-NC EPCs; no luminal structures or cells in Matrigel implants without cells were observed (Figure 2B). To confirm our *in vivo* results with EPC function and angiogenesis, we next investigated the role of *GUSBP5-AS* in angiogenesis in EPCs *in vitro*. The tube formation of *GUSBP5-AS*-overexpressing EPCs was significantly increased compared with the LV5-NC group, while angiogenesis in GUSBP5-AS-knockdown EPCs was attenuated compared to the LV3-NC group (Figure 2C). These results demonstrated that *GUSBP5-AS* is functionally involved in EPC-associated angiogenesis. To examine the role of *GUSBP5-AS* in cell migration, we performed wound healing assays and observed that migration of EPCs with *GUSBP5-AS*-knockdown was significantly lower than that of the LV3-NC group while the migration ability of the *GUSBP5-AS* overexpression group was significantly greater than that of the LV5-NC group (Figure 2D). Transwell invasion assay showed similar results; inhibition of *GUSBP5-AS* significantly reduced cell invasion, while overexpression of *GUSBP5-AS* enhanced cell invasion (Figure 2E). We further studied whether *GUSBP5-AS* acts on cell cytoskeleton that is closely related to changes in cell migration capacity. As shown in Figure 2F, downregulation of *GUSBP5-AS* impaired F-actin filaments while *GUSBP5-AS* overexpression prevented disruption of the actin cytoskeletal structure. These results revealed that GUSBP5-AS plays a central role in the migration and invasion ability of EPCs in vitro, likely by regulating the expression of F-actin, at least in part.

### *GUSBP5-AS* have a central role in EPC growth

**Figure 3:**
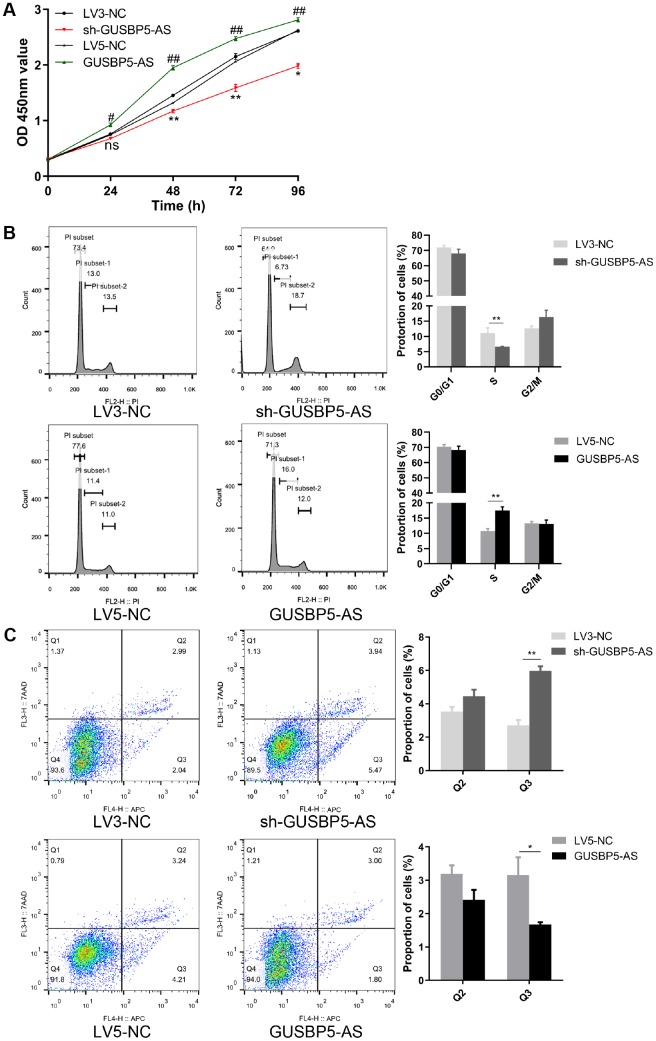
***GUSBP5-AS* have a central role in proliferation.** (**A**) Cell proliferation ability of *GUSBP5-AS* in lncRNA *GUSBP5-AS* knockdown and overexpression group and their corresponding NC groups was detected by CCK8 assay. *P< .05, **P< .01 compared to the LV3-NC group; ^#^P < .05, ^##^P < .01 compared to the LV5-NC group. (**B**) The effect of *GUSBP5-AS* on EPC cycle was investigated by FCM cell cycle distribution test. (**C**) The effect of lncRNA *GUSBP5-AS* on cell apoptosis was measured with Annexin V-APC/7-AAD Apoptosis Detection Kit by FCM. Cell apoptosis was analysed. Data from 3 experiments are summarized. *P< .05, **P< .01 compared to the corresponding negative control group. Data are represented as mean ± SEM.

We next performed CCK8 assays to evaluate the role of GUSBP5-AS in regulating EPC proliferation. Cell proliferation was increased in the GUSBP5-AS overexpression group compared with controls, while the proliferative ability of EPCs in the GUSBP5-AS-knockdown group was decreased compared with the LV3-NC group (Figure 3A). Changes in cell proliferation are usually associated with changes in cell cycle progression. To study the mechanism by which *GUSBP5-AS* regulates EPC proliferation, we performed cell cycle analysis on EPCs with *GUSBP5-AS* overexpression and knockdown and their corresponding control groups. Flow cytometry analysis revealed that overexpression of *GUSBP5-AS* increased the proportion of EPCs arrested in G1/S phase but decreased the portion in G2/M phase compared with the LV5-NC group. *GUSBP5-AS* inhibition significantly reduced the proportion of cells in S phase compared with the LV3-NC group (Figure 3B). These results suggested that *GUSBP5-AS* exerts an important role in cell cycle regulation, especially in S phase. We next investigated whether *GUSBP5-AS* regulates apoptosis in EPCs. Flow cytometric analysis of treated EPCs showed that *GUSBP5-AS* overexpression significantly decreased the percentage of total apoptotic cells, while inhibition of *GUSBP5-AS* significantly promoted apoptosis, especially for early apoptotic cells (Figure 3C). Taken together, our data demonstrated that *GUSBP5-AS* has a critical function in the regulation of *in vivo and in vitro* angiogenesis of EPCs, migration, invasion, and proliferation, and inhibits cell apoptosis.

### *GUSBP5-AS* is closely related to the regulation of EPC function by RNA sequencing

**Figure 4:**
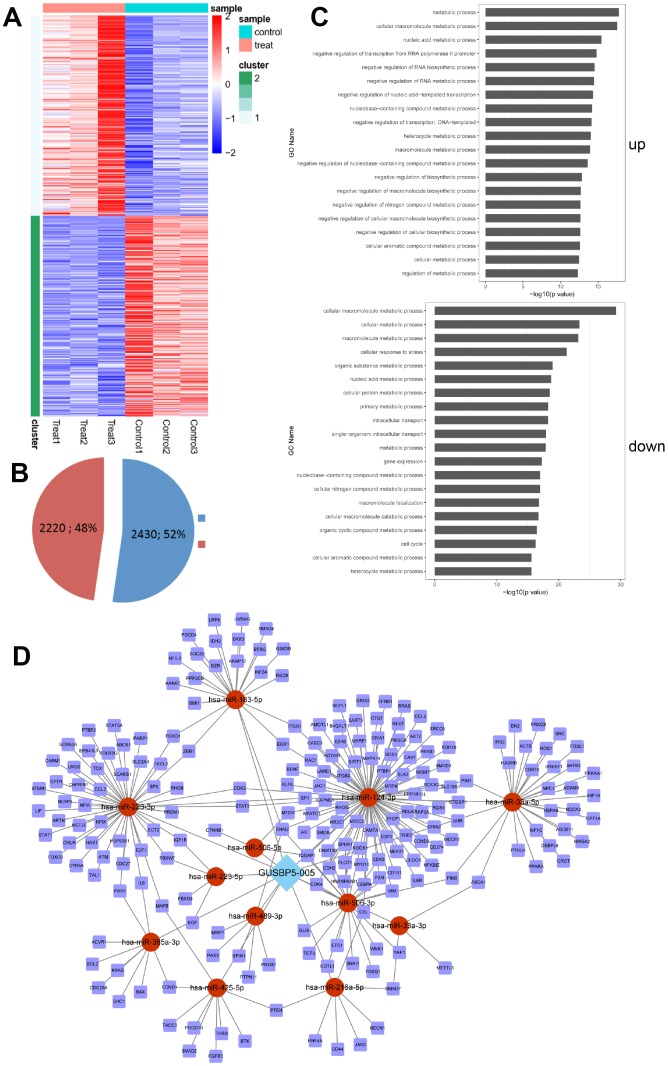
**The up- and down- regulated mRNAs were filtrated using microarray analysis.** (**A**) Heatmap analysis of dysregulated mRNAs in negative controls and *GUSBP5-AS*-overexpressing EPCs. Control group indicates LV5-NC group and treat for *GUSBP5-AS*-overexpressing EPC group. (**B**) Proportion of up- and down- regulated genes were filtered using microarray analysis in the *GUSBP5-AS* overexpression group compared with the LV5-NC group. Blue indicates up-regulated genes, and red for down-regulated genes. (**C**) Several biological process pathways were enriched. Up indicates the enrichment analysis of up-regulated genes, and down for down-regulated genes. (**D**) The regulatory network of lncRNA-miRNA-mRNA.

For validation purposes, we next performed RNA sequencing (RNAseq) to evaluate gene expression profiles in *GUSBP5-AS*-overexpressing EPCs by microarray analysis. mRNA expression levels were screened in *GUSBP5-AS*-overexpressing EPCs and negative controls using hierarchical clustering analysis (Figure 4A). The results showed that 2,430 (52%) genes were upregulated and 2,220 (48%) genes were downregulated in the *GUSBP5-AS* overexpression group compared with the control group (Figure 4B). To identify relevant biological functions, Gene Ontology (GO) enrichment analysis of Biological Processes (GOBP) was performed. Genes modulated by *GUSBP5-AS* showed a significant involvement mainly in categories related to metabolic processes, stress, cell cycle, and gene expression (Figure 4C). Following verification of the regulatory role of *GUSBP5-AS* *in vivo* and *in vitro*, we next investigated the underlying mechanism of *GUSBP5-AS* in the modulation of angiogenesis and migration in EPCs. As shown in Figure 4D, lncRNA-miRNA-mRNA network showed that 12 predicted miRNAs including *miR-223-3p* may be targets of *GUSBP5-AS*. Based on the results of mRNAseq and the regulatory network of lncRNA-miRNA-mRNA, we used qRT-PCR to detect the expression levels of all the predicted target miRNAs (Data were not shown), and found that *miR-223-3p* expression was significantly decreased by *GUSBP5-AS* whereas downregulation of *GUSBP5-AS* significantly increased the expression levels of *miR-223-3p*. Moreover, the main genes involved in angiogenesis and migration that are significantly altered by *GUSBP5-AS* are shown in Table 1 and Supplementary Figure 1. The results of RNAseq and qRT-PCR suggested that the main factors, FOXO1, MMP2, MMP9 and FGF2, were upregulated and inhibited, respectively, by *GUSBP5-AS* overexpression and knockdown.

**Table 1:** RNAseq analysis results of the main factors involved in angiogenesis and migration regulated by *GUSBP5-AS* overexpression.

**Gene_id**	**Log2FoldChange**	**Stat**	**p value**	**p adj**	**Significant**
MMP9	1.680414704	4.19413	2.74E-05	0.000111	Ups
FGF2	1.381545685	3.44619	0.000569	0.001783	Ups
MMP2	1.22681	2.06987	0.0008	0.005605	Ups
FOXO1	1.232851707	5.50857	3.62E-08	2.35E-07	Ups

RNAseq analysis was performed after EPCs infection with LV5-NC and *GUSBP5-AS* lentivirus, and three groups of LV5-NC and three groups of *GUSBP5-AS* were detected; Gene id means differentially expressed genes; p value was calculated by t test, and p adj was obtained by p value normalized by Benjamini-Hochberg; significant means the up- or down-regulation of the gene expressed in the *GUSBP5-AS* overexpression group compared with the LV5-NC.

### *GUSBP5-AS* directly binds *miR-223-3p* to regulate migration and angiogenesis in EPCs

**Figure 5:**
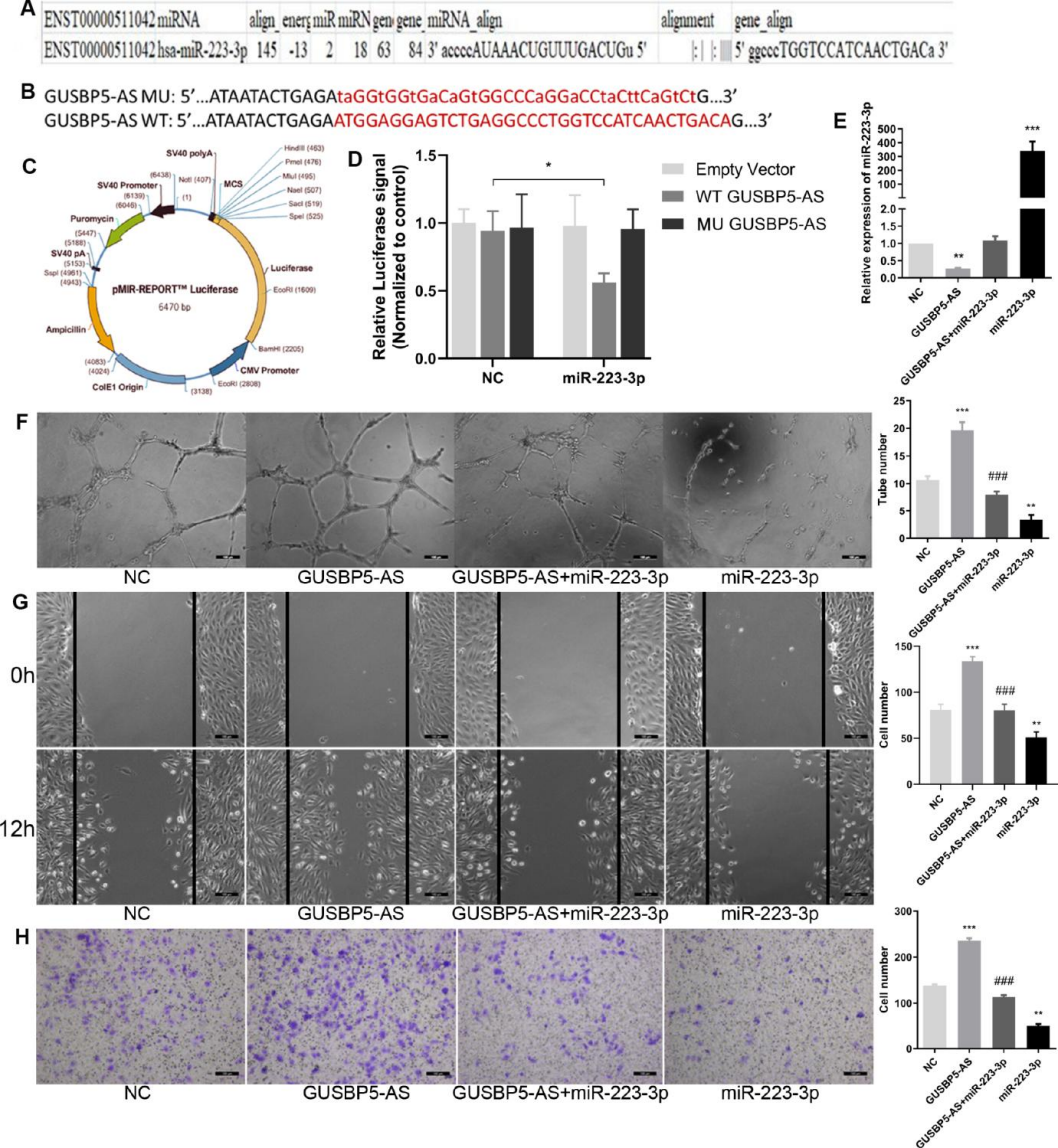
**LncRNA *GUSBP5-AS* directly bound with *miR-223-3p*.** (**A**) Bioinformatics analysis revealed *GUSBP5-AS* contains binding sequences complementary to the binding sites of *miR-223-3p*. (**B**) The luciferase reporter constructs containing WT-*GUSBP5-AS* or MU-*GUSBP5-AS* sequence. (**C**) Schematic graph of the constructed reporter plasmid containing putative. (**D**) Dual-luciferase reporter assay verified the targeting relationship between *miR-223-3p* and *GUSBP5-AS*. (**E**) The expression of *miR-223-3p* was measured by qRT-PCR after cells treated with *GUSBP5-AS*, *GUSBP5-AS*+*miR-223-3p* or *miR-223-3p*. (**F**) Effects of cotransfection of *GUSBP5-AS* and *miR-223-3p* on in vitro tube formation were analysed. Scale bar=100μm. (original magnification, ×100). (**G**) Effects of cotransfection of *GUSBP5-AS* and *miR-223-3p* on cell migration were detected by wound healing assay. Scale bar=100μm. (original magnification, ×100). (**H**) Effects of cotransfection of *GUSBP5-AS* and *miR-223-3p* on cell invasion were analysed by transwell cell invasion assay. Scale bar=100μm. (original magnification, ×100). **P < .01, ***P < .001 compared to the NC group, ^###^P < .001 compared to the *GUABP5-AS* treated group. Data are represented as mean ± SEM.

To further verify the binding relationship between *miR-223-3p* and *GUSBP5-AS*, bioinformatics prediction tools was used. These data also revealed that *miR-223-3p* has complementary binding sites that target the 3’ untranslated region (3’ UTR) of *GUSBP5-AS* (Figure 5A). Dual luciferase reporter assay confirmed that *miR-223-3p* binding to *GUSBP5-AS* displayed reduced luciferase activity, indicating molecular interactions (Figure 5B–5D). These data indicated that *GUSBP5-AS* directly sponges *miR-223-3p*. As shown in Figure 5E, *GUSBP5-AS* suppressed *miR-223-3p* expression, and the expression of *miR-223-3p* increased following overexpression of *miR-223-3p*. These results indicated that *miR-223-3p* is a downstream molecular target of *GUSBP5-AS*. To further explore the function of *GUSBP5-AS* and *miR-223-3p* in EPCs, migration, invasion, and tube formation experiments were performed. Overexpression of *GUSBP5-AS* and *miR-223-3p* in EPCs significantly increased and decreased tube formation, migration and invasion, respectively, compared with the NC group (Figure 5F–5H). We did not observe differences between the *GUSBP5-AS* and *miR-223-3p* mimics cotransfection group and the NC group.

### FOXO1 is a direct target of *miR-223-3p* in EPCs

**Figure 6:**
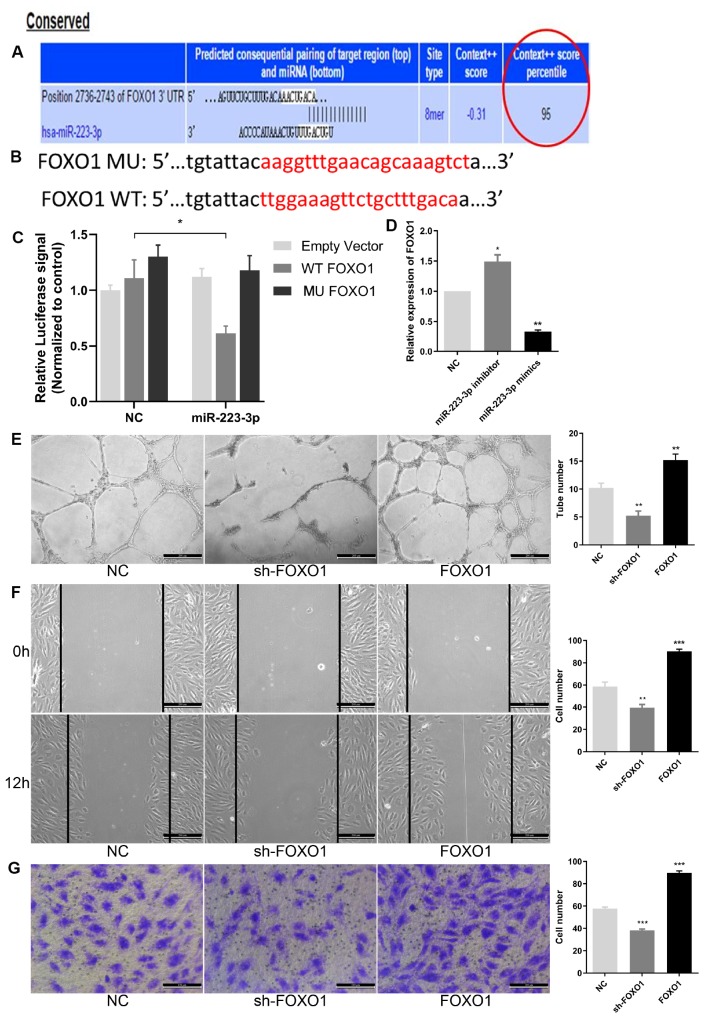
**FOXO1 acted as the direct target of *miR-223-3p*.** (**A**) Bioinformatics programs assay revealed complementary binding within *miR-223-3p* and FOXO1 3’UTR. (**B**) The luciferase reporter constructs containing WT-FOXO1 or MU-FOXO1 sequence. (**C**) Dual-luciferase reporter assay verified the targeting relationship between miR-223-3p and FOXO1. (**D**) Relative quantification of mRNA levels of FOXO1. Three independent experiments were performed. (**E**) Tube formation assay showing the angiogenesis ability of EPCs by FOXO1 knockdown and overexpression. Scale bar=200μm. (original magnification, ×100). FOXO1 knockdown and overexpression, respectively, repressed and enhanced capability of angiogenesis in EPCs. (**F**) Effects of FOXO1 on cell migration were analysed by wound healing assay. Scale bar=200μm. (original magnification, ×100). FOXO1 knockdown and overexpression, respectively, reduced and increased number of EPCs migrated. (**G**) Transwell invasion assay showed that FOXO1 knockdown and overexpression, respectively, inhibited and promoted the ability of cell invasion in EPCs. Scale bar=100μm. (original magnification, ×200). **P < .01, ***P < .001 compared to NC group. Data are represented as mean ± SEM.

To investigate the mechanism of *miR-223-3p* in angiogenesis, the downstream target genes of *miR-223-3p* were predicted using the Starbase, TargetScan, miRanda, and miRDB databases. According to RNAseq analysis, the regulatory network map and predicted targets of *miR-223-3p* (Table 1 and Figure 4D), FOXO1, playing a key role in cell function, may be a potential target gene of *miR-223-3p*, and the binding sequences between *miR-223-3p* and FOXO1 mRNA are shown in Figure 6A. FOXO1 is a member of the FOX transcription factor family, which is involved in numerous crucial cellular processes including metabolism, proliferation, and migration, as well as cell cycle, apoptosis, and angiogenesis progression [34, 35]. Previous studies showed that FOXO1 expression can be regulated by miRNAs. To examine the association between *miR-223-3p* and FOXO1, luciferase reporter plasmids of *miR-223-3p* and FOXO1 were used (Figure 6B). Reduced luciferase reporter activity was observed after cotransfection of the luciferase reporter plasmid containing WT-FOXO1 and *miR-223-3p* mimics in HEK-293T cells (Figure 6C). Moreover, *miR-223-3p* mimics downregulated FOXO1 mRNA expression while *miR-223-3p* increased its expression in EPCs (Figure 6D). Furthermore, to further verify the correctness of our results, we tested the role of FOXO1 in the regulation of EPC function. As showed in Figure 6E–6G, knockdown of FOXO1 significantly inhibited the abilities of EPC angiogenesis, migration and invasion, whereas FOXO1 overexpression obviously enhanced EPC function. These results indicated that *miR-223-3p* targets and negatively regulates FOXO1 in EPCs.

### The relationship between *GUSBP5-AS*, *miR-223-3p* and FOXO1 in EPCs from DVT patients

**Figure 7:**
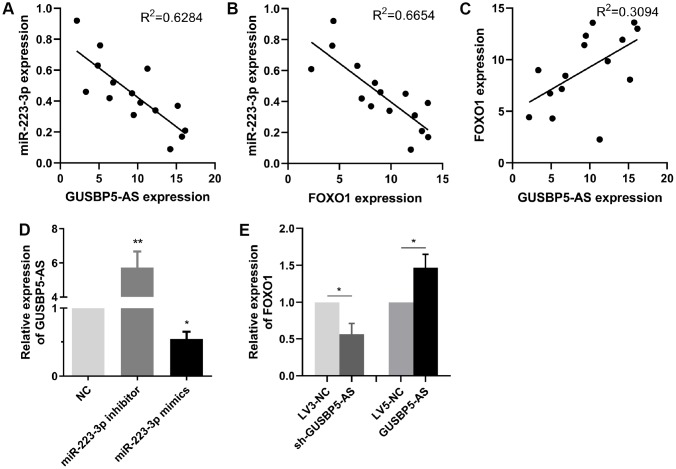
**The relationship between *GUSBP5-AS*, *miR-223-3p* and FOXO1 expression.** (**A**–**C**) Spearman rank correlation analyses showed negative correlation with *GUSBP5-AS* and *miR-223-3p* in 15 cases of EPCs samples from DVT patients, and FOXO1 was negatively correlated with *miR-223-3p* expression, while *GUSBP5-AS* was positively correlated with FOXO1 expression. (**D**) *miR-223-3p* mimics and inhibitor, respectively, inhibited and enhanced the expression of *GUSBP5-AS* in EPCs. (**E**) *GUSBP5-AS* knockdown and overexpression, respectively, reduced and increased the mRNA expression levels of FOXO1 in EPCs. *P < .05, **P < .01 compared to the corresponding control group. Data are represented as mean ± SEM.

As shown in Figures 5E and 6D, *GUSBP5-AS* overexpression significantly downregulated *miR-223-3p* expression and *miR-223-3p* inhibitor observably increased the expression levels of FOXO1 in EPCs by qRT-PCR assays. To test the relationship between *GUSBP5-AS*, *miR-223-3p* and FOXO1 in EPC samples from DVT patients, the Spearman rank correlation analysis were performed by qRT-PCR. The data showed that *miR-223-3p* expression was negatively correlated with *GUSBP5-AS* and FOXO1 expression, while *GUSBP5-AS* expression was positively correlated with FOXO1 expression (Figure 7A–7C). Additionally, we verified the expression levels of *GUSBP-AS* and FOXO1 in EPCs infected with *miR-223-3p* and *GUSBP-AS*, respectively. The results showed that *miR-223-3p* mimics and inhibitor significantly suppressed and enhanced *GUSBP5-AS* expression respectively, and the expression of FOXO1 was notably decreased and increased, respectively, by *GUSBP5-AS* knockdown and overexpression, compared to the corresponding controls (Figure 7D–7E).

### Role of *GUSBP5-AS* and *miR-223-3p* in the regulation of the FOXO1/Akt pathway, FGF2 and MMP2/9 expression

**Figure 8:**
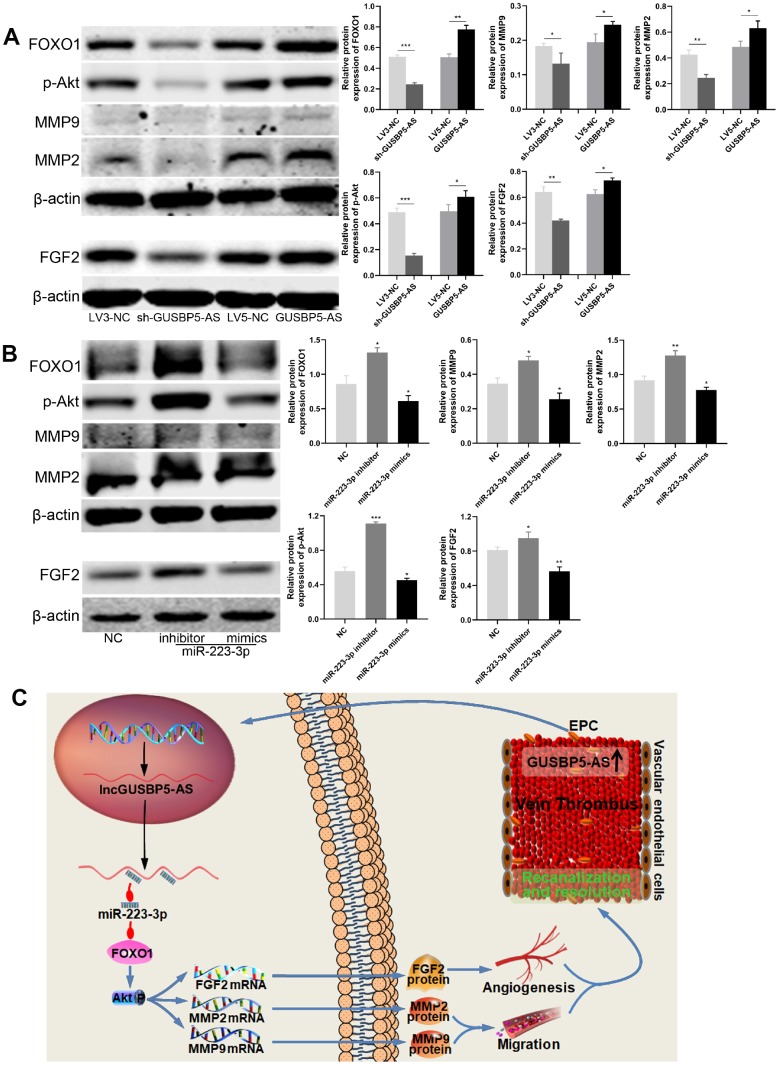
***GUSBP5-AS* regulates FGF2 and MMP2/9 expression through FOXO1/Akt pathway by sponging with *miR-223-3p*.** (**A**) Effects of *GUSBP5-AS* on the regulation of the protein expression of FOXO1, p-Akt, MMP2, MMP9 and FGF2 in EPCs by western blot analysis. *GUSBP5-AS* knockdown and overexpression, respectively, reduced and increased these protein expression levels. (**B**) Western blot analysis of the protein expression levels of FOXO1, p-Akt, MMP2, MMP9 and FGF2 in EPCs after infected with NC, *miR-223-3p* inhibitor and mimics. The resulted showed that *miR-223-3p* inhibitor and mimics, respectively, promoted and inhibited these protein expression. *P < .05, **P < .01, ***P < .001 compared to the corresponding control group. Data are represented as mean ± SEM. (**C**) Schematic of the role of *GUSBP5-AS*-mediated effects in EPCs. Red arrow indicates inhibition and blue arrow indicates promotion.

To further explore the effects of *GUSBP5-AS* on intracellular pathways and the factors related to angiogenesis and migration, the protein expression levels of FOXO1, p-Akt, FGF2, MMP2 and MMP9 were detected by western blot, indicating that *GUSBP5-AS* knockdown significantly reduced these protein levels, while *GUSBP5-AS* overexpression increased them (Figure 8A). Importantly, our data showed that *miR-223-p* inhibitor enhanced FOXO1, p-Akt, FGF2, MMP2 and MMP9 expression whereas *miR-223-p* mimics suppressed their expression levels (Figure 8B). Interestingly, according to the previous study, FOXO1 can feed back to Akt and activate Akt pathway [36]. Thus, our results revealed that *GUSBP5-AS* played a central role in the regulation of angiogenesis *in vivo* and *in vitro*, migration and invasion ability of EPCs by regulating the expression levels of FGF2, MMP2 and MMP9 through *miR-223-3p*/FOXO1/Akt pathway.

## DISCUSSION

In this study, we demonstrated that *GUSBP5-AS* promotes EPC angiogenesis *in vitro* and *in vivo* and accelerates EPC homing to thrombus sites to promote DVT recanalization and resolution. *In vitro*, overexpression of *GUSBP5-AS* promoted EPC proliferation, migration, invasion and angiogenesis, and inhibited cell apoptosis. Inhibition of its expression had the opposite effects. These findings greatly extend our current understanding of the role of lncRNAs in DVT, clarifying a novel mechanism for *GUSBP5-AS* in EPCs and providing a new target for the management of DVT.

DVT is a common and difficult problem in the clinic. Complications such as PE and PTS caused by DVT are life-threatening [4, 5, 9]. Thrombus revascularization is an important event in the resolution of DVT [12, 13]. Thus, it is very important and necessary to develop alternative therapies to accelerate thrombus revascularization and resolution. Increasing evidence has demonstrated that EPCs have a critical role in the angiogenesis and resolution of DVT. However, the limited capacities of homing and angiogenesis impair the therapeutic effect of EPCs. Thus, it is very important to solve these limitations to improve their clinical application.

Intriguingly, recent studies have revealed that specific lncRNAs can regulate angiogenesis and development of vascular diseases [21, 37, 38]. We previously showed that *GUSBP5-AS*, located at chr4q31.21, is upregulated in EPCs from DVT patients compared with those from healthy controls according to microarray analysis and was predicted to be a crucial regulator in angiogenesis [32]. As shown in Figure 8C, herein we explored the effect and mechanism of *GUSBP5-AS* in EPCs and DVT. The results demonstrated that the up-regulated *GUSBP5-AS* in DVT significantly reduced thrombus size and weight, effectively accelerated EPC homing to thrombi to promote thrombus recanalization and resolution. Additionally, the mRNA-seq analysis showed *GUSBP5-AS* was involved in metabolic processes, stress, cell cycle, and gene expression, which is closely related to cell proliferation, migration and angiogenesis. Further, our results demonstrated that *GUSBP5-AS* promoted EPC proliferation, angiogenesis *in vitro* and *in vivo*, migration, invasion, F-actin filaments, FGF2 and MMP2/9 expression, and decreased apoptosis. Knockdown of *GUSBP5-AS* had the opposite effects. Actin and the microtubule system are required for cell adhesion and migration [39], and FGF2 and MMP2/9 are essential factors for promoting EPC migration, angiogenesis and early venous thrombus resolution [40–42]. The results suggested that *GUSBP5-AS* enhanced EPC proliferation, angiogenesis, migration and invasion via promoting FGF2, MMP2/9 and F-actin expression, at least in part.

MiRNAs have been demonstrated to be dysregulated in human diseases and may act as sensitive and specific biomarkers in certain vascular diseases including DVT, arteriosclerosis, aneurysm, and pulmonary arterial hypertension [43]. Growing evidence has confirmed that miRNAs have a vital role in the regulation of proliferation, migration, invasion, angiogenesis, cell cycle progression and apoptosis. *MiR-223* has been shown to reduce proliferation, migration, and angiogenesis in cancer cells and endothelial cells [44, 45]. However, the role and mechanism of *miR-223-3p* in the regulation of EPCs and DVT has not been examined. In this study, we demonstrated the target relationships between *GUSBP5-AS* and *miR-223-3p* and between *miR-223-3p* and FOXO1, and we confirmed their interaction by correlation analysis. The data showed that *GUSBP5-AS* and FOXO1 were overexpressed in EPCs of DVT patients. Conversely, the expression level of *miR-223-3p* is significantly downregulated in EPCs samples of DVT patients. Moreover, our results showed that co-transfection of *GUSBP5-AS* and *miR-223-3p* further confirmed the effects of their interaction on EPC migration, invasion and tube formation, and FOXO1 knockdown and overexpression, respectively, inhibited and enhanced the abilities of angiogenesis, migration and invasion in EPCs. Together, our results suggested that the *GUSBP5-AS*/*miR-223-3p*/FOXO1 axis appears to play a crucial role in DVT progression and EPC angiogenesis, providing a novel biomarker and promising therapeutic target for DVT patients.

Notably, FGF2, a potent angiogenic factor, regulates a wide variety of functions and plays angiogenic and anti-apoptotic roles in vascular endothelial cells [46]. The matrix metalloproteinase (MMP) superfamily is composed of metalloproteinases that degrade extracellular matrix, a necessary process prior to cell migration, invasion and angiogenesis [47, 48]. Among these factors, MMP2 and MMP9 play an essential role in vascular disease [40, 49]. Previous studies have demonstrated that FGF2 and MMP2/9 can be up-regulated by activation of Akt phosphorylation [46, 47, 50]. Akt is a vital regulator of several physiological and pathological conditions, including cell proliferation, migration, angiogenesis and autophagy [50, 51]. Importantly, FOXO1, one of the most widely studied members in FOXO family, is a critical rheostat of vascular expansion, endothelial metabolism and growth [34], and deletion of *Foxo1* (*Foxo1 ^EC-KO^*) resulted in defective vascular development [34], and FOXO1 dependent pathways have been a research hotspot for the past decade [52]. Previous studies have found that FOXO1 can feed back to Akt and promote activation of Akt in endothelial cells [36]. In the present study, our results showed that FOXO1 was the target of *miR-223-3p* and promoted activation of Akt pathway, which enhanced the expression of F-actin, FGF2, MMP2 and MMP9, subsequently regulating EPC migration, angiogenesis and resulation of DVT.

In conclusion, our study substantiated that *GUSBP5-AS* was significantly upregulated and modulated *miR-223-3p*/FOXO1 axis in EPCs of DVT patients. *GUSBP5-AS* promotes cell angiogenesis, proliferation, migration and invasion in EPCs by regulating F-actin filaments, FGF2, MMP2 and MMP9 expression through the *miR-223-3p*/FOXO1/Akt pathway, thereby promoting EPC homing to thrombi and enhancing thrombosis recanalization and resolution. These findings may provide a novel biomarker and potential therapeutic target for patients with DVT.

## MATERIALS AND METHODS

### Patients and specimens

A total of fifteen pairs of peripheral blood samples from patients with a first life-time acute DVT confirmed by ultrasonic doppler and healthy controls were obtained at The Second Affiliated Hospital of Soochow University between September 2017 and April 2018 and used to culture EPCs. None of the patients had undergone surgery or received anticoagulant drugs before blood collection. The age of DVT patients was 34-75 years, and the mean age was 54.45±15.01 years. And the age of these healthy controls was 35-72 years, and the mean age was 53.12±14.52 years. The inclusion criteria were as follows: i) Swelling, pain, and superficial varicose veins in the lower limbs and other clinical manifestations for acute DVT; and ii) DVT confirmed by ultrasound doppler. Exclusion criteria for the two groups were: i) people who had hypertension, diabetes, cardiovascular disease, liver and kidney dysfunction, or severe infection and ii) people with diseases such as immune system disease or active bleeding and other diseases. In this study, all subjects or their families signed informed consent. EPCs were identified by confocal microscopy and flow cytometry, as previously described [21].

### Animals

Male nude mice were purchased from Shanghai Lingchang Biotechnology Co., Ltd. (Shanghai, China) and rasied in the Institutional Animal Care and Use Committee of Soochow University, presented in a pathogen-free facility with constant room temperature, humidity and light cycle (12:12 h light: dark), and free food and water.

### Cell transfection

All oligonucleotides were purchased from GenePharma (Shanghai, China), including recombinant lentiviral particles expressing the short hairpin RNAs (shRNAs) sh-lncRNA *GUSBP5-AS* (targeting *GUSBP5-AS*), *GUSBP5-AS*, *miR-223-3p* mimics, *miR-223-3p* inhibitor, and their corresponding negative controls. And the sequence (5’ to 3’) of sh-*GUSBP5-AS* is AGCATGAACTTCACGTCAAGA. Lentiviruses were used to infect EPCs according to the manufacturer’s instructions and polybrene (5 μg/mL) was used to enhance infection efficiency. Cells were then selected by 3 μg/mL puromycin (Invivogen, San Diego, CA, USA) for 1 week to construct stable *GUSBP5-AS* or *miR-223-3p* overexpression and knockdown cell lines along with controls.

### Analysis of the therapeutic effect on deep vein thrombosis

Nude mice (10 weeks old) were anesthetized with 1% pentobarbital sodium at 10 μL/g body weight. The left jugular vein of the nude mouse was isolated under a stereo microscope, and the proximal end of the heart and its branches were ligated. GFP-*GUSBP5-AS* EPCs and GFP-LV5-NC EPCs (5×10^6^) were injected into the nude mice through the orbital vein. After 7 days, thrombi with vessel walls were harvested for measurements of size and weight, and for immunofluorescence. The number of GFP-EPCs at the thrombus was observed under an IX-81 laser confocal microscope (Olympus) to analyze homing ability. To immunostain CD34 and MMP2, frozen sections were stained with anti-CD34 (abcam, Cambridge, UK; ab81289, 1:100) or anti-MMP2 (abcam, ab92536, 1:200). After incubation with fluorescent-labeled secondary antibodies (donkey anti-rabbit Alexa Fluor® 647, abcam, 1:500) at room temperature for 60 min, images were observed with a confocal microscope.

### *In vivo* angiogenesis assay

200μL Matrigel (BD), and Matrigel mixture containing EPCs (5×10^5^) infected with *GUSBP5-AS*, sh-*GUSBP5-AS* and corresponding negative controls were implanted on the flank of the 5-week-old male nude mice. After 7 days, Matrigel implants were removed, fixed in 4% paraformaldehyde, embedded in paraffin, and then sectioned. Histological sections were stained with hematoxylin and eosin (H&E) to detect the presence of luminal structures. Images were obtained by an Olympus DP73 microscope (Tokyo, Japan). Microvascular structures were counted in three high-power views (200× magnification) of each section.

### Matrigel tube formation assay

Transfected EPCs (5×10^4^ cells) were seeded in Matrigel-coated wells of a 24-well plate to induce tube formation. After 12 h of incubation, the tubular structures of EPCs in the Matrigel were examined by microscopy, and the extent of tube formation was assessed by measuring the tube number and tube length with ImageJ software. At least five random fields (100× magnification) per well were examined to determine cell tube formation ability. All experiments were repeated three times.

### Wound healing assay

Wound healing migration assays were performed with EPCs in 6-well plates. When the cells had grown to nearly 90% confluency, the cell monolayer was scratched using a 200 μL pipette tip to create a wound. After removing the cellular debris with PBS, the cells were incubated with serum-free EBM-2 medium for a specific duration. Cell migration was observed using an inverted microscope and cells were imaged at 0 and 12 h after the scratch. The wound size was quantified using Image J in three wells per group.

### Transwell assay

Cell invasion assays were performed using 24-well Matrigel chambers (8-mm pore size; BD, USA) containing 12 μL Matrigel-coated membranes. Each cell suspension in serum-free EBM-2 medium was counted by microscopy. Medium containing 10% FBS (300 μL) was added to the lower wells. Each group of cells (2×10^4^ cells in 200 μL serum-free EBM-2 medium) was added to the upper transwell chambers. Matrigel chambers were incubated at 37°C in a 5% CO_2_ incubator for 24 h.

### Proliferation assay

Cell proliferation was measured using a Cell Counting Kit-8 (CCK-8). The transfected EPCs were seeded into 96-well plates. After 0, 24, 48, 72 and 96 h, the medium was replaced with 100 μL detection buffer (ratio of medium and CCK-8, 9:1) and cells were incubated for 2 h. The absorbance at 450 nm was detected by a microplate reader (Bio-Rad). Experiments were performed in triplicate.

### Cell cycle assay

Cells were harvested by trypsinization and fixed with 70% ethanol at 4°C overnight. The fixed cells were washed with PBS and successively incubated with propidium iodide (PI; 50 μg/mL) and RNase A (100 μg/mL) for 1 h at room temperature, followed by analysis by flow cytometry. All tests were performed in triplicate.

### Apoptosis assay

Cell apoptosis was analyzed using a BD Accuri™ C6 flow cytometer (BD Biosciences, USA) and an Annexin V-APC/7-AAD Apoptosis Detection Kit (KeyGEN BioTECH), according to the manufacturer’s instructions. Flow cytometric analysis of 7-AAD-Annexin V staining was repeated at least three times.

### Immunofluorescence

Transfected EPCs were grown on cover slides for 24 h. Cells were washed with PBS and then fixed with 4% paraformaldehyde for 10 min at room temperature. After three washes, the cells were blocked with Immunol Staining Blocking Buffer (Beyotime, P0102) for 1 h at room temperature. Immunostaining was performed using TRITC Phalloidin (Solarbio, CA1610) to identify F-actin. Cell nuclei were stained with DAPI. The coverslips were washed with PBS and inverted onto slides that had been treated with Fluoromount-GTM seal. Images were acquired by confocal microscopy under the same conditions for each experiment, and no digital manipulation of images was performed.

### RNAseq assay

Total RNA was extracted from 1×10^7^ cells using RNAiso Plus (TaKaRa). RNAseq was performed using EPCs stably expressing empty vector (LV5-NC) or *GUSBP5-AS*. An RNA library was generated according to Illumina’s RNAseq protocol. The samples were fragmented and primed for cDNA synthesis, adapters were ligated to the cDNA, and the resulting samples were amplified by PCR. Sequencing was performed on Illumina HiSeq 2000 paired-end module (Illumina).

### Luciferase activity assay

For luciferase reporter assays, the 3’UTR segments of *FOXO1* predicted to interact with *miR-223-3p* were amplified by PCR. Cells were cotransfected with firefly luciferase reporter plasmid (lncRNA *GUSBP5-AS*-WT, lncRNA *GUSBP5-AS*-MU, FOXO1-WT, FOXO1-MU) and a *Renilla* luciferase vector (pMIR-Report Luciferase, Promega) plus negative control and small RNAs (NC, *miR-223-3p* mimics) using Lipofectamine 2000 (Invitrogen, USA). Experiments were performed at least three times. Cells were harvested 48 h later, and firefly and *Renilla* luciferase activities were analyzed with a Modulus™ single tube multimode reader (Sunnyvale, CA, USA).

### Quantitative reverse transcriptase-PCR (qRT-PCR) analysis

Total RNA was extracted from harvested cells using RNAiso Plus (TaKaRa, Otsu, Japan), and first-strand cDNA synthesis was performed using a PrimeScript RT reagent kit (TaKaRa). qRT-PCR analysis was carried out using SYBR Green quantitative PCR (qPCR) Master Mix (Bio-Rad, CA, USA). MiRNA levels were measured using the Hairpin-it™. miRNAs RT-PCR Quantitation Kit (GenePharma) according to the manufacturer’s instructions. mRNA expression levels were analyzed using mRNA primers (GENEWIZ, Jiangsu, China). The endogenous control for *miR-223-3p* was *U6* RNA and *GAPDH* for the others. qRT-PCR was performed on a High Throughput Quantitative PCR LightCycler480 II (Roche, Basel, Switzerland). The relative expression levels of each candidate gene were calculated using the 2^^-ΔΔ CT^ method [24]. Each experiment was performed independently and in triplicate. The qRT-PCR primers are shown in Supplementary Table 1.

### Western blot analysis

Transfected cells were cultured for 48 h. Total cellular protein was isolated with RIPA buffer (50 mM Tris-HCL pH 7.4, 150 mM NaCl, 1%NP-40, 0.5% sodium deoxycholate, and 0.1% SDS) supplemented with protease inhibitors, phosphatase inhibitors, and PMSF. Cellular debris was removed by centrifugation at 12000 g for 10 min at 4°C. Protein quantification was performed using a BCA Protein Assay Kit (Beyotime, P0011). Lysates were separated by 10% or 15% SDS PAGE gels and transferred to PVDF membranes (Bio-Rad). Membranes were subjected to western blotting using antibodies against FOXO1 (Cell Signaling, 1:1000), p-Akt (Cell Signaling, 1:1000), FGF2 (Cell Signaling, 1:1000), MMP2 (Abcam, 1:1000), MMP9 (Abcam, 1:1000) and β-actin (Cell Signaling, 1:1000). IRDye 800CW goat anti-rabbit or anti-mouse IgG (Licor) was used at 1:10,000 dilution. Infrared Imagine System (LI-COR) was performed to analyze proteins.

### Statistical analysis

In all *in vitro* studies, three separate experiments were performed on cells from different transfection experiments. Data are presented as mean ± standard error of the mean (SEM). Student’s *t*-test was used for comparing means between two groups. Statistical significance was analyzed by one-way ANOVA in three or more groups using GraphPad Prism 8 software. *P*-value of 0.05 or less was considered statistically significant.

## Supplementary Material

Supplementary Figure 1

Supplementary Table 1
