# Supplementary material for: LncRNA GUSBP5-AS promotes EPC migration and angiogenesis and deep vein thrombosis resolution by regulating FGF2 and MMP2/9 through the miR-223-3p/FOXO1/Akt pathway
Source: Aging (Albany NY). 2020 Mar 10;12(5):4506–26. doi: 10.18632/aging.102904 (PMC7093182; doi:10.18632/aging.102904)
Supplement: Supplementary Figure 1 [file aging-12-102904-s002..pdf]

SUPPLEMENTARY FIGURE

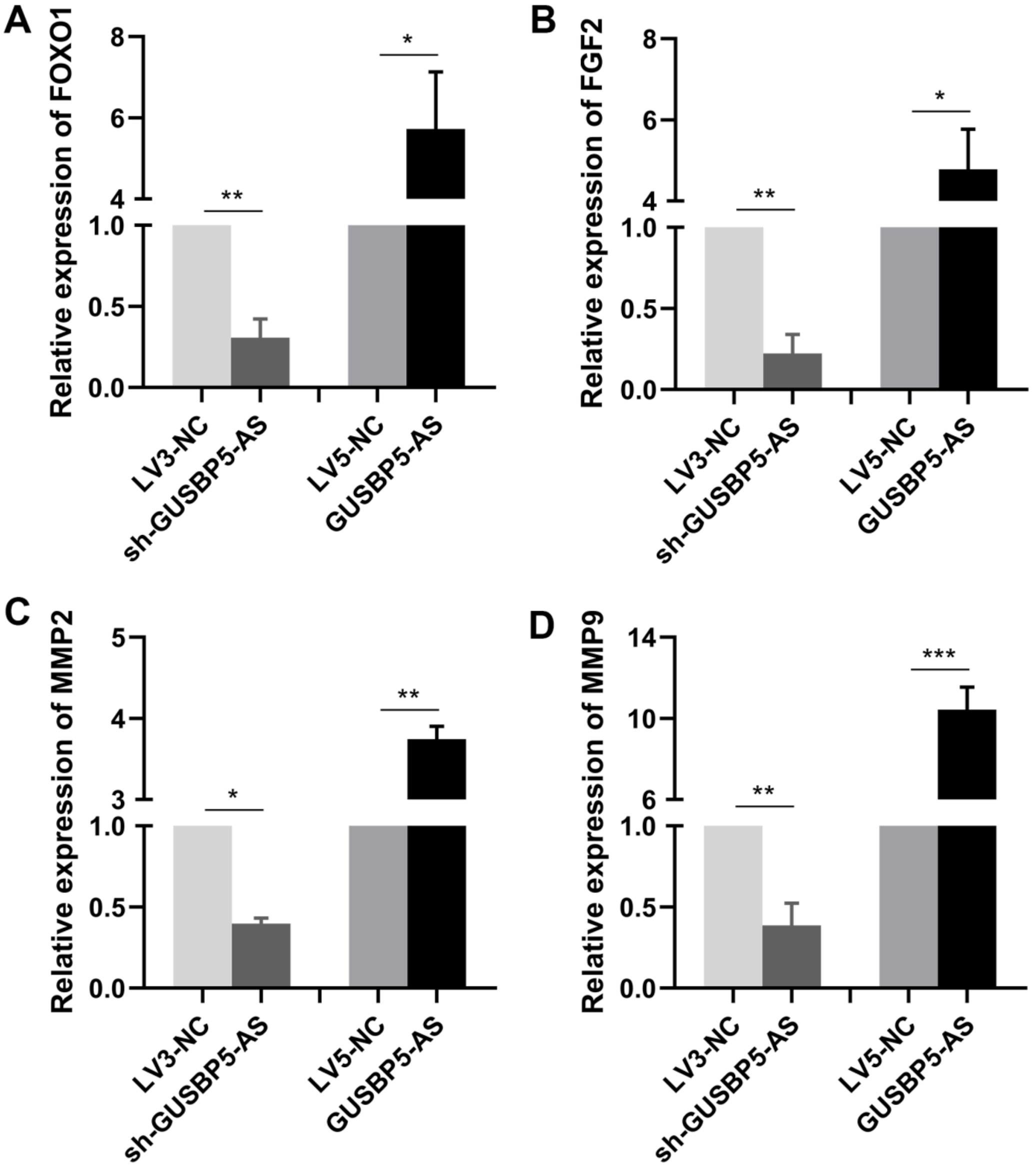

Supplementary Figure 1. qRT-PCR analysis of the main angiogenesis and migration-related genes. (**A**–**D**): Relative quantification of mRNA levels of FOXO1, FGF2, MMP2 and MMP9 were determined by qRT-PCR in EPCs infected with sh-*GUSBP5-AS* and *GUSBP5-AS*. *P < .05, **P < .01, ***P < .001 compared to the corresponding control group.
