# Supplementary material for: LncRNA GUSBP5-AS promotes EPC migration and angiogenesis and deep vein thrombosis resolution by regulating FGF2 and MMP2/9 through the miR-223-3p/FOXO1/Akt pathway
Source: Aging (Albany NY). 2020 Mar 10;12(5):4506–26. doi: 10.18632/aging.102904 (PMC7093182; doi:10.18632/aging.102904)
Supplement: Supplementary Table 1 [file aging-12-102904-s001..pdf]

SUPPLEMENTARY TABLE

Supplementary Table 1. Primers used for qRT-PCR.

Has-GUSBP5-AS	Forward (5′---3′)	CTGGTCCATCAACTGACAGCA
	Reverse (5′---3′)	CAGAGCAGGGTTCATACATCC
Has-FOXO1	Forward (5′---3′)	TCGTCATAATCTGTCCCTACACA
	Reverse (5′---3′)	CGGCTTCGGCTCTTAGCAAA
Has-MMP9	Forward (5′---3′)	TGTACCGCTATGGTTACACTCG
	Reverse (5′---3′)	GGCAGGGACAGTTGCTTCT
Has-MMP2	Forward (5′---3′)	TGACTTTCTTGGATCGGGTCG
	Reverse (5′---3′)	AAGCACCACATCAGATGACTG
FGF2	Forward (5′---3′)	AGAAGAGCGACCCTCACATCA
	Reverse (5′---3′)	CGGTTAGCACACACTCCTTTG
Has-miR-223-3p	Forward (5′---3′)	GTTGCTCCTGTCAGTTTGTCAAA
	Reverse (5′---3′)	TATGGTTGTTCACGACTCCTTCAC
Has-GAPDH	Forward (5′---3′)	CATGAGAAGTATGACAACAGCCT
	Reverse (5′---3′)	AGTCCTTCCACGATACCAAAGT
Has-U6	Forward (5′---3′)	CTCGCTTCGGCAGCACA
	Reverse (5′---3′)	AACGCTTCACGAATTTGCGT
